# A versatile 3D culture model facilitates monitoring of astrocytes undergoing reactive gliosis

**DOI:** 10.1002/term.209

**Published:** 2009-12

**Authors:** Emma East, Jonathan P Golding, James B Phillips

**Affiliations:** Department of Life Sciences, The Open UniversityMilton Keynes, UK

**Keywords:** CNS injury, glial scar, 3D culture model, GFAP, CSPG, vimentin, aquaporin 4, TGFβ1

## Abstract

A major impediment to CNS repair is the glial scar, which forms following damage and is composed mainly of ramified, ‘reactive’ astrocytes that inhibit neuronal regrowth. The transition of astrocytes into this reactive phenotype (reactive gliosis) is a potential therapeutic target, but glial scar formation has proved difficult to study in monolayer cultures because they induce constitutive astrocyte activation. Here we demonstrate a 3D collagen gel system in which primary rat astrocytes were maintained in a persistently less reactive state than comparable cells in monolayer, resembling their status in the undamaged CNS. Reactivity, proliferation and viability were monitored and quantified using confocal, fluorescence and time-lapse microscopy, 3D image analysis, RT–PCR and ELISA. To assess the potential of this system as a model of reactive gliosis, astrocytes in 3D were activated with TGFβ1 to a ramified, reactive phenotype (elevated GFAP, Aquaporin 4, CSPG, Vimentin and IL-6 secretion). This provides a versatile system in which astrocytes can be maintained in a resting state, then be triggered to undergo reactive gliosis, enabling real-time monitoring and quantitative analysis throughout and providing a powerful new tool for research into CNS damage and repair. Copyright © 2009 John Wiley & Sons, Ltd.

## 1. Introduction

Traumatic injuries to the brain and spinal cord are debilitating and often lead to cognitive impairment, paralysis and loss of sensation. Failure of the injured CNS to repair is in part attributed to the inhibitory environment of the lesion site, most notably the formation of the glial scar, which consists predominantly of astrocytes and forms a physical and physiological barrier to axon regeneration (Fawcett and Asher, 1999). Astrocytes in the undamaged CNS express low levels of glial fibrillary acidic protein (GFAP) (Cancilla *etal.*, 1992) but, following injury, exhibit a reactive hypertrophic phenotype (reactive gliosis) exemplified by upregulation of various markers, including GFAP, vimentin, aquaporin-4 (AqP4), S100β and chondroitin sulphate protoglycans (CSPGs) (Calvo *etal.*, 1991; Hirsch and Bahr, 2000; Silver and Miller, 2004; Saadoun *et al.*, 2005; Neal *et al.*, 2007). A common finding of strategies aimed at bridging CNS lesions, particularly tissue-engineered approaches using biomimetic materials (Phillips *et al.*, 2004), is that although axons readily enter and traverse the bridging graft, they seldom exit the graft and re-enter the host parenchyma, due to the inhibitory glial scar at the graft-CNS interface (Geller and Fawcett, 2002).

Research in this field often focuses on the physiology of CNS injury in experimental animals. Using these *in vivo* models is an essential stage in developing new strategies for treating human CNS damage, but these models are often too complex for the isolation and control of specific variables and, when seeking to understand cell-level biology, they allow only a snapshot view upon post mortem examination. For many research questions in neuroscience, in particular exploring the behaviour of specific cell populations involved in damage and repair or establishing mechanisms involved in glial scar development and maturation, *in vitro* models can provide a valuable tool. Commonly used *in vitro* models of the glial scar have employed mechanical scraping of two-dimensional (2D) astrocyte cultures to produce a ‘wound’ (Yu *et al.*, 1993), stretching of astrocytes cultured on silastic membranes (Ellis *et al.*, 1995; Wanner *et al.*, 2008) and creation of a glial scar *in vivo* on nitrocellulose membranes that are removed and cultured *in vitro* (McKeon *et al.*, 1991). Whilst these and other 2D culture models have revealed key information regarding the reactivity of astrocytes and their effects on neuronal growth (reviewed by (Wu and Schwartz, 1998)), there are limitations to their usefulness for studying the process of reactive gliosis, since astrocytes in 2D cultures are highly reactive, making it difficult to monitor further activation.

Scientists in other areas of biology are increasingly utilizing three-dimensional (3D) cell culture systems, which allow researchers to investigate cell behaviour in a more physiologically relevant state (Lee *et al.*, 2008). Furthermore, the glial scar is a 3D structure composed of a meshwork of interwoven astrocytic processes that form a barrier, which consequently is very difficult to model in 2D cultures (Fawcett *et al.*, 1989). Tissue-engineered 3D culture models offer an exciting opportunity to fill the gulf in nervous system research between simple cell culture systems and whole-animal models (Pampaloni *et al.*, 2007; Lee *et al.*, 2008). These can provide a valuable means to investigate cell biology, using highly controlled environments in which parameters are easily manipulated to gain insight into the fundamental biological processes that follow nervous system injury whilst maintaining the cells in a meaningful spatial arrangement.

The aim of this work was to develop a 3D culture system in which astrocytes could be maintained in a less reactive manner than in conventional monolayer culture. For such a system to be useful, it would be reproducible, controllable and amenable to a wide variety of analyses, including immunocytochemistry, time-lapse and confocal microscopy, ELISA and RT-PCR. Furthermore, astrocytes would be capable of responding to a damage stimulus in a manner similar to their *in vivo* counterparts (changes in morphology, expression of proteins and release of soluble factors).

To achieve these aims, type-I collagen was chosen as the substrate of choice for 3D cultures, due to the ease with which it can be controlled and manipulated to enable versatile monitoring and imaging. It is a good basal substrate that can be used as a scaffold on which cells can deposit additional extracellular matrix (ECM) molecules as they modify their 3D environment. This is particularly important for investigating proteoglycan synthesis by astrocytes in response to activation, which would be difficult to detect in more complex 3D culture substrates (e.g. Matrigel™), which often contain mixtures of a wide range of ECM proteins from the outset.

To test the effectiveness of this approach, the viability and activity of astrocytes in 3D collagen gels was investigated, then they were treated with TGFβ1, considered to be a likely trigger for astrogliosis *in vivo* (Silver and Miller, 2004), causing astrocyte responses typical of reactive gliosis. Thus we report a model in which reactive gliosis can be controlled and monitored to improve the development of CNS repair strategies.

## 2. Materials and methods

### 2.1. Primary cortical astrocyte cultures

All experiments were performed according to the UK Animals (Scientific Procedures) Act (1986) and approved by the appropriate institutional ethical committee. Sprague-Dawley rats were used from established in-house breeding colonies. Primary astrocyte cultures were prepared from the cortices of postnatal 2 day-old rat pups (adapted from Dutton *et al.*, 1981). Following decapitation, the cortices were dissected out and the meninges and associated blood vessels removed with fine forceps. The tissue was roughly chopped with a scalpel blade, placed in 250 μg/ml trypsin in 10 ml disaggre-gation medium [14 mM glucose (Sigma, Dorset, UK), 3 mg/ml bovine serum albumin (BSA; Sigma), 1.5 mM MgSO_4_ (BDH, VWR, UK) in Ca^2+^- and Mg^2+^-free Earle's balanced salt solution (Gibco, Invitrogen, Paisley, UK)] for 15 min at 37 °C, and agitated every few minutes. Following this incubation, a dilute solution of soya bean trypsin inhibitor (21 μg/ml SBTI; Sigma) and deoxyri-bonuclease 1 (6 μg/ml DNase; Sigma) was added and the cell suspension was centrifuged for 2 min at 250 × g to pellet the tissue. The supernatant was removed and the pellet resuspended in 500 μl concentrated solution of SBTI and DNase (133 μg/ml and 40 μg/ml, respectively). The solution was triturated using a 1 ml pipette to break down any larger clumps of tissue; a further 500 μl concentrated SBTI and DNase solution was added and the suspension left to settle for 2 min. The material at the top of the suspension was removed to a separate 15 ml tube. This trituration procedure was repeated twice more. The resulting cell suspension was then underlain with 4% w/v BSA, which was then centrifuged at 250 × g for 5 min. The supernatant was gently removed and the pellet resuspended in Dulbecco's modified Eagle's medium (DMEM; Gibco) supplemented with penicillin/streptomycin (100 U/ml and 100 μg/ml respectively; Sigma) and with 10% v/v fetal calf serum (FCS). This suspension was dispensed into 75 cm^2^ flasks (Greiner, Stonehouse, UK) that had been coated with poly-D-lysine (Sigma) at an approximate density of 1-2 cortices/flask. Each flask was topped up with 20 ml DMEM and placed in a humidified incubator at 37 °C with 5% CO_2_:95% air.

### 2.2. 2D and 3D astrocyte cultures

Astrocytes were expanded in culture for 8 days to reach confluence. The flasks were shaken at 150 rpm for 4 h to deplete the microglia and less adherent cells from the cultures. Following shaking, the resulting cultures were 95% astrocytes (as determined by immunoreactivity for GFAP and IB4 lectin). The medium was removed and 7 ml trypsin-EDTA solution (Sigma) was added to each flask for 15 min at 37 °C. The trypsin was neutralized by the addition of 13 ml DMEM/flask, supplemented with penicillin/streptomycin and 10% FCS. The cells were centrifuged at 250 × g for 5 min and the pellet resuspended in 1 ml medium; the cells were counted using a haemocytometer. For 2D astrocyte cultures, coverslips were coated with 15 μg type I rat tail collagen (First Link UK Ltd, Birmingham, UK) in 0.6% acetic acid for 1 h. This solution was removed and the coverslips were then rinsed with sterile phosphate-buffered saline (PBS) to removed any traces of acid, then were left to dry at room temperature. 35 K astrocytes were plated in 100 μl suspension per collagen-coated coverslip in multiwell plates and left to adhere for 1 h at 37 °C before adding 2 ml DMEM supplemented with penicillin/streptomycin 10% FCS/well.

For 3D astrocyte cultures, cells were seeded at a density of 2 million cells/ml gel. The gels were composed of 10% cell suspension in DMEM, 10% lOx minimum essential medium (MEM; Sigma) and 80% type-I rat tail collagen (2mg/ml in 0.6% acetic acid; First Link, UK). The MEM and collagen were mixed together and neutralized using sodium hydroxide, assessed by colour change of phenol red. Upon neutralization, the collagen-MEM mixture was gently mixed with the cell suspension and transferred to 24-well plates (0.75 ml/well; resulting gels approximately 4 mm thick) before placing at 37 °C to set (∼5 min). Once the gels had set, the wells were topped up with 2 ml DMEM supplemented with penicillin/streptomycin and 10% FCS. Coverslips and gels for experiments comparing 2D and 3D conditions were maintained in culture for 24 h before fixing or RNA extraction.

### 2.3. Cell viability and proliferation

Cell viability was assessed using propidium iodide (PI; Sigma) staining in combination with Hoechst. Briefly, PI was added to cultures at 200 μg/ml in cell culture medium and left to incubate for 10 min at 37 °C. The medium was then removed and the cultures were rinsed in PBS before fixing in 4% paraformaldehyde (PFA) at 4 °C. Gels and coverslips were incubated with Hoechst 33 258 (1 μg/ml; Sigma) in PBS for 10 min, before 3x5 min washes in PBS. Bromodeoxyuridine (BrdU) incorporation was used to assess cell proliferation in addition to cell counts. BrdU was added into cell culture medium at 10 μM and incubated at 37 °C for 20 min. The cultures were washed twice with DMEM, then PBS, before fixing in 4% PFA. For BrdU only, coverslips and gels were incubated with 0.5% Triton-X-100 to permeabilize the cells before treating with DNase at 37 °C for 1 h. Detection of BrdU was performed as described in the Immunocytochemistry section below. The number of dead or proliferating cells was calculated as a percentage of the total number of cells per field (three randomly selected fields per coverslip or gel).

### 2.4.RT-PCR

Total RNA was isolated from primary astrocytes using Trizol® (Invitrogen). For 2D cultures, the medium was removed and Trizol was added directly onto the cells. The cells were then scraped and the resulting solution collected into sterile 2 ml tubes. The medium was removed from 3D gels; following a wash in sterile PBS, each gel was added to a sterile 2ml tube containing 1 ml Trizol, which was then frozen in liquid nitrogen. After thawing, the gels were homogenized in the Trizol by trituration with a 19 G hypodermic needle on a syringe until no lumps of collagen gel were remaining. RNA extraction was then carried out according to the manufacturer's instructions. The yield of total RNA was quantified by optical density (OD) readings at 260 nm, and the purity was estimated by the 260 :280 nm ratio.

Equivalent amounts of total RNA (3 μg) from astrocyte preparations were reverse-transcribed into single-stranded cDNA in a reaction mixture containing 10 mM dithiothreitol, 40 U RNase inhibitor, 10 mM Tris-HCl, pH 8.3, 15 mM KCl, 0.6 mM MgCl_2_, 0.5 mM dNTPs, 250 ng random primers and 600 U MMLV reverse transcriptase (Invitrogen) at 37 °C for 80 min. Incubation for 10 min at 70 °C terminated the reverse transcription reaction. Negative controls were prepared by incubation of samples without reverse transcriptase.

PCR was performed on the equivalent of 100 ng reverse-transcribed total RNA from each sample. A ther-mocycler was used with a reaction mixture containing 50 mM KCl, 10 mM Tris-HCl, pH 9, 0.1% Triton X-100, 0.5 mM MgCl_2_, 200 μM dNTPs, 1 μM each upstream and downstream primer, and 2.5 U TaqDNA polymerase (Invitrogen). MgCl_2_ concentrations and optimal annealing temperatures (OAT) were as follows: (1) GFAP (McKeon *etal.*, 1999), 1.5 mM MgCl_2_, OAT 60 °C, 20 cycles, forward 5'-GCCGCTCCTATGCCTCCTCCGA-3', reverse 5'-TCCAGCGACTCAACCTTCCTCT-3'; (2) GAPDH (Copelman *et ah*, 2000), 2 mM MgCl_2_, OAT 60 °C, 25 cycles, forward S'-TGGTGCCAAAAGGGTCATCATCTCC-S', reverse S'-GCCAGCCCCAGCATCAAAGGTG-S'; (3) Neurocan (Qi *etal.*, 2003), 1.5 mM MgCl_2_, OAT 60°C, 20 cycles, forward 5'-CTGCTTCTTTACCCTTCAACCAC-3', reverse S'-AGTTGTCAAAGCCATCTTCGAAC-S'. Primer pairs spanned an exon boundary to obviate DNase treatment of total RNA before reverse transcription (RT)-PCR. PCR performed on non-reverse-transcribed total RNA samples did not produce any detectable products. The thermal cycle profile for each set of primers included a primary denaturation cycle at 94 °C for 5 min and a final extension period at 72 °C for 10 min. The intervening PCR cycle consisted of 45 s segments of primer denaturation, annealing and extension.

Equivalent amounts (12 μl) of each PCR product were size-fractionated on a 1% agarose gel and the product size was verified by running the samples against a 100 bp DNA ladder (Promega, Southampton, UK). The PCR products were visualized by staining the agarose gels with 0.5 μg/ml ethidium bromide and viewing under ultraviolet light.

### 2.5. Time-lapse microscopy

Imaging was performed using an Olympus IX70 inverted microscope with heated stage set at 37°C and a 20× viewing objective. Images were captured using In Vivo 3 software (Media Cybernectics, MD, USA), then processed using Volocity image analysis software (Improvision, Perkin-Elmer, Coventry, UK). For time-lapse microscopy, 2D and 3D astrocyte cultures were prepared as described above. Cells were plated onto collagen-coated coverslips or seeded into collagen gels and were viewed for 24 h, with images taken every 10 min. The cultures were kept in CO_2_-independent medium (Gibco) for the duration of the time-lapse experiment.

### 2.6. Astrogliosis model

3D astrocyte gels were treated with transforming growth factor-β 1 (TGFβ1; 10 ng/ml; R&D Systems, Abingdon, UK), diluted in DMEM supplemented with penicillin/streptomycin and 10% FCS every other day for 15 days. TGFβ1 has previously been used as a positive reactivity control (Cullen *et al.*, 2007) and induces specific alterations that are consistent with astrogliosis (Logan *etal.*, 1994). Furthermore, TGFβ1 has been identified as one of the potential triggers of inhibitory astrogliosis (Silver and Miller, 2004). Control gels were subject to media change only on the same days. Supernatants were collected throughout the experiments for subsequent analysis of cytokines and gels were fixed at days 1, 5, 10 and 15 throughout the experiment.

### 2.7. Immunocytochemistry

The same procedure for immunocytochemistry was performed on coverslips and gels, with the incubation and wash times increased for gels. After fixing, the gels were cut into quarters for staining and image analysis. Cell permeabilization was performed using 0.5% Triton-X-100 (Sigma), 15 min for coverslips, 30 min for gels. Following 3 × 5 min washes, non-specific binding was blocked with 5% normal swine serum (Dako, Ely, UK) in PBS for 20 min (coverslips) or 40 min (gels). After another wash step, primary antibodies were diluted in PBS (Table 1) and incubated at room temperature for 1.5 h (coverslips) or overnight at 4°C (gels). Following 3 × 10 min washes, secondary antibodies [anti-rabbit fluorescein isothiocyanate (FITC), anti-rabbit tetramethylrhodamine isothiocyanate (TRITC), anti-mouse FITC or anti-mouse TRITC (Sigma)] were diluted in PBS (1 in 100) and added for 45 min (coverslips) or 1.5 h (gels). Hoechst 33 258 (1 μg/ml) was also added into the secondary antibody dilutions for cell counting. Omission of primary or secondary antibody was routinely used as a control. Coverslips were placed on slides in fluorescence mounting medium (Dako) and sealed with nail varnish. Gels were stored in PBS.

**Table 1:** Primary antibodies used in immunocytochemistry

Primary antibody	Dilution	Source
BrdU	1 in 500	Abcam clone BU1/75
GFAP	1 in 300	Dako
Vimentin	1 in 50	Sigma clone V9
CSPGs	1 in 100	Sigma clone CS-56
AqP4	1 in 300	Chemicon
S100β	1 in 1000	Dako
β-Actin	1 in 200	Sigma clone AC15

### 2.8. Image analysis and quantification

Fluorescence microscopy was performed on coverslips and gels for comparison between 2D and 3D cultures. Images were captured using an Olympus BX61 microscope with Analysis® Pro imaging software (Olympus Soft Imaging System, Munster, Germany). Three fields were randomly selected per coverslip or gel. The number of immunopositive cells was calculated as a percentage of the total number of cells/field. In addition, the average number of cells/field was compared between astrocytes in gels for 1 and 24 h as another indication of cell proliferation.

Confocal microscopy (Leica DMIRBE, Leica Microsystems, Mannheim, Germany) was performed on control gels and gels treated with TGFβ1 and images were captured using Leica confocal software (Leica, Germany); each field measured 1 × 1 mm × 40 μm (*xyz*), with 40 slices/stack in the *Z* dimension. Analysis included 14 independent gels per time point (seven control and seven TGFβ1), from two separate cell preparations. Three fields per gel quarter were selected according to a robust protocol (2 mm in from all edges in a triangular formation). The perimeter of cell staining (GFAP, AqP4, Vimentin) and the area of staining (CS56) were calculated using Volocity image analysis software (Improvision, Perkin-Elmer). Automated analysis protocols were developed by identifying cells by intensity of staining, so various measurements could be calculated.

Cell shape was analysed using Openlab (Improvision) software. Extended-focus GFAP images captured by confocal microscopy were analysed, with three fields per gel and seven gels per time point per condition (i.e. control and TGFβ1 treatment). The formula utilized for this analysis by the software is (4 × jr A)/P^2^, where *A* is the cell area and *P* is the cell perimeter. A perfectly spherical cell will have a value of 1, whilst smaller values indicate progressively more irregularly shaped cells, thus providing an indicator of how rounded or ramified (i.e. with branch-like processes) the cells were.

### 2.9. Enzyme-linked immunosorbant assay (ELISA) for interleukin 6 (IL-6)

A quantitative sandwich ELISA for IL-6 (R&D Systems) was carried out according to the manufacturer's instructions. Briefly, a standard curve for rat IL-6 was prepared (range 0-4000 pg/ml). 50 μl standard, sample (cell culture supernatant), blank or positive control were added to a 96-well plate precoated with rat-specific IL-6 capture antibody. After incubation for 2 h at room temperature, the plate was washed and the horseradish peroxidise-conjugated detection antibody was incubated for 2 h at room temperature. Following further washing, a tetram-ethlybenzidine substrate solution was added to the wells and incubated for 30 min at room temperature in the dark. The enzyme reaction was stopped using dilute hydrochloric acid and the OD of each well was read at 450 nm, with reference reading at 570 nm.

### 2.10. Statistical analysis

Data were analysed with the GraphPad Prism computer package (GraphPad Software, San Diego, CA, USA). Normality and quality of variance tests were performed on all data to determine which test was appropriate. A t-test was used, with significance level set at 95% for comparison between 2D and 3D culture datasets and to compare between control and TGFβ1-treated cultures at each of the time points. If variances of datasets were significantly different, then Welch's correction was applied. A one-way ANOVA with Dunnett's multiple comparison *post hoc* test was performed to compare control data points over the 15 day experimental period. All values are indicated as the mean ± standard error of the mean (SEM). *p* values were taken as an indicator of statistical significance, using the following nomenclature: *p < 0.05, **p < 0.01 and ***p < 0.001.

## 3. Results

### 3.1. Astrocytes in 3D cultures are constitutively less activated than in 2D

**Figure 1:**
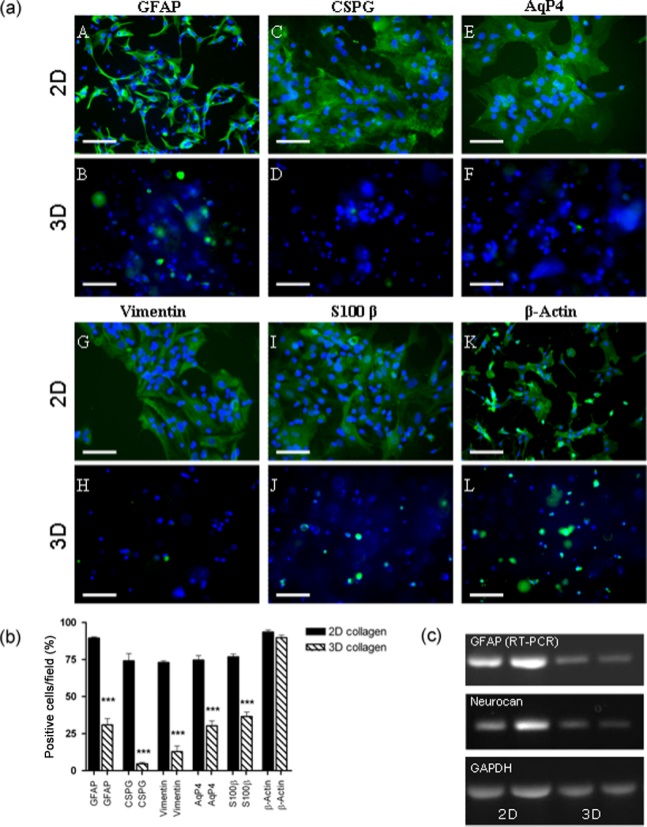
Astrocytes in 3D collagen gels have lower expression of activation markers. (a) Immunofluorescent staining for activation markers GFAP (A + B), CS56 (C + D), aquaporin 4 (E + F), vimentin (G + H) and S100β (I + J) were compared in 2D astrocyte cultures on collagen-coated coverslips and in 3D collagen gels. Staining for β-actin (K + L) was used as a positive control. Hoechst 33258 was used to detect cell nuclei. Scale bar = 100 μm. (b) Staining for all activation markers was significantly decreased on astrocytes in 3D collagen gels compared to astrocytes in 2D cultures. Staining for β-actin was comparable for both 2D and 3D astrocyte cultures (data are mean ± SEM; *n* = 7; ***p < 0.001). (c) RT–PCR showed lower levels of mRNA for GFAP and neurocan in astrocytes from 3D collagen gel cultures when compared to astrocytes grown in monolayers. Representative samples shown

Astrocytes on collagen-coated coverslips (2D) or in collagen gels (3D) were compared for immunofluorescence staining (Figure 1a) of the activation markers GFAP (A + B), CSPGs (C + D), AqP4 (E + F), vimentin (G + H) and S100β (I + J) after 24 h in culture. The number of astrocytes immunopositive for all of the activation markers in 3D cultures were significantly fewer when compared to astrocytes grown on 2D monolayer cultures (Figure 1b; *p* < 0.001). To confirm that this result was not due to differences in staining protocol or reduced diffusion of antibodies through the gels, staining for *β-*actin was carried out as a positive control (Figure 1a; K + L). The number of β-actin-immunopositive astrocytes did not differ significantly between 2D and 3D cultures, thus indicating that the differences in the activity markers was due to astrocytes in 3D gels exhibiting a less reactive phenotype than those in 2D, after 24 h.

In order to investigate whether mRNA levels of GFAP and an astrocyte-specific CSPG, neurocan, were affected by changes in cell culture dimension, in addition to protein levels, RT-PCR was performed for these markers and compared to the house-keeping gene *GAPDH.* Message levels for neurocan and more notably GFAP were reduced in astrocytes cultured in 3D collagen gels, compared to those grown in 2D monolayers (Figure 1c).

### 3.2. Cell viability and proliferation

**Figure 2:**
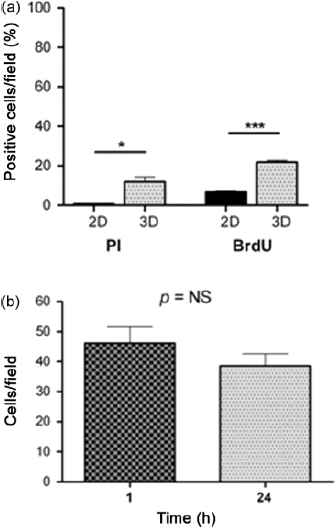
Cell death and proliferation in 2D and 3D astrocyte cultures. (a) Cell death and proliferation of astrocytes was compared between 2D and 3D cultures using PI staining and BrdU incorporation at a 24 h time point. The number of cells immunopositive for PI and BrdU was significantly higher in astrocytes in 3D collagen gels than those on collagen-coated coverslips. (b) The total number of astrocytes per field after 1 h and 24 h did not differ significantly (data are mean ± SEM; *n* = 4; *p < 0.05, ***p < 0.001)

Staining for PI and BrdU incorporation was used as a way of determining cell death and proliferation in 2D and 3D astrocyte cultures. The percentage of PI-positive and BrdU-positive cells was significantly greater in astrocytes cultured in 3D collagen gels than those growing on coverslips after 24 h (Figure 2a; *p* < 0.05 andp < 0.001, respectively). To determine the extent of astrocyte proliferation in 3D culture over 24 h, astrocytes were seeded into collagen gels and left for 1 and 24 h, then the total numbers of cells/field were calculated. The number of cells did not significantly change between 1 and 24 h in culture, suggesting no astrocyte proliferation (Figure 2b). Thus, the BrdU-positive cells observed after 24 h could indicate cells that had undergone S-phase but not yet progressed through G2 and M phases prior to cell division. The 2D PI data may not truly represent cell death, since dead cells in monolayer culture tend to float off the coverslips into the medium. Nevertheless, small differences in cell death and/or proliferation between the culture dimensions cannot entirely account for the large differences observed in activation markers in Figure 1c.

### 3.3. Time-lapse microscopy - differences in cell morphology and motility between 2D and 3D astrocyte cultures

**Figure 3:**
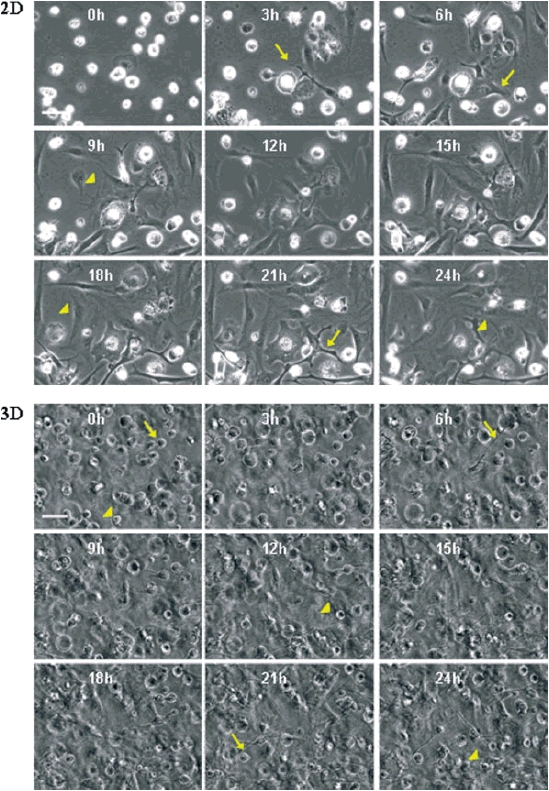
Time-lapse imaging of the initial 24 h of 2D and 3D astrocyte cultures. Astrocytes were seeded onto coverslips or into collagen gels and were imaged using time-lapse microscopy for the initial 24 h in culture. Images were taken every 10 min; representative images are shown from 0, 3, 6, 9, 12, 15, 18, 21 and 24 h time points (see Supporting information for two time-lapse movies). The key differences observed were that cells in 2D had larger cell area and flattened morphology (arrowheads) with wider filopodia (arrows) compared to astrocytes in 3D. Astrocytes in 3D extended fine filopodia (arrows) and migrated through the gel, but were generally rounder and had a smaller cell area (arrowheads). Scale bar = 100 μm

One of the major benefits to *in vitro* approaches is the ability to monitor cells in real time. To investigate the behaviour of astrocytes in our 3D collagen gel system, cultures were subject to time-lapse microscopy over the initial 24 h following seeding and compared to conventional 2D astrocyte cultures. Representative images from 3 h time periods are shown in Figure 3 and two time-lapse movies are available in the Supporting information. Astrocytes in 2D monolayer spread and migrated over the initial 24 h in culture. In general, these astrocytes had larger cell area and flattened morphology (Figure 3, arrowheads, 2D; 9, 18 and 24 h), with wider filopodia (Figure 3, arrows, 2D; 3, 6 and 21 h) compared to astrocytes in 3D. Astrocytes in 3D extended thin filopodia that could project over distances up to 400 *\im* (Figure 3, arrows, 3D; 0, 12 and 24 h). Furthermore, astrocytes in 3D cultures were able to migrate easily through the gel matrix but appeared more round in shape and smaller than astrocytes in 2D cultures (Figure 3, arrowheads, 3D; 0, 6 and 21 h).

### 3.4. Induction of astrogliosis byTGFβ1 treatment

**Figure 4:**
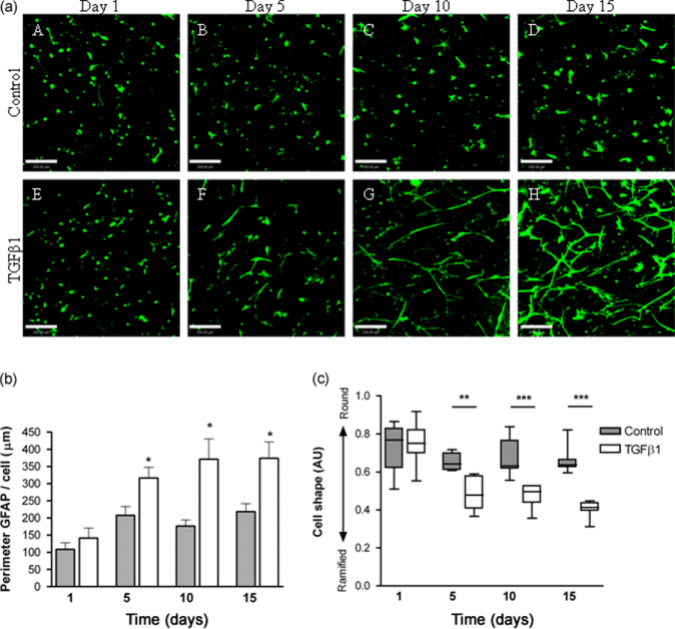
Perimeter of astrocytic GFAP staining increases with TGFβ1-treatment. (a) Representative extended focus confocal images of control (A–D) and TGFβ1-treated (E–H) astrocytes over 15 days in culture stained for GFAP. Scale bar = 200 μm. (b) Quantification of GFAP staining showed that cell perimeters significantly increased in TGFβ1-treated astrocytes from 5 days in culture compared to non-treated control cultures at the same time point. Data are mean ± SEM normalized to cell number (*n* = 7; *p < 0.05). (c) Shape analysis of GFAP-positive cells, whereby values close to 1 represent perfect circular cells and values < 1 indicate irregular, ramified cells. Cells from TGFβ1-treated cultures were significantly more ramified than controls after 5, 10 and 15 days in culture (*n* = 7; **p < 0.01, ***p < 0.001)

**Figure 5:**
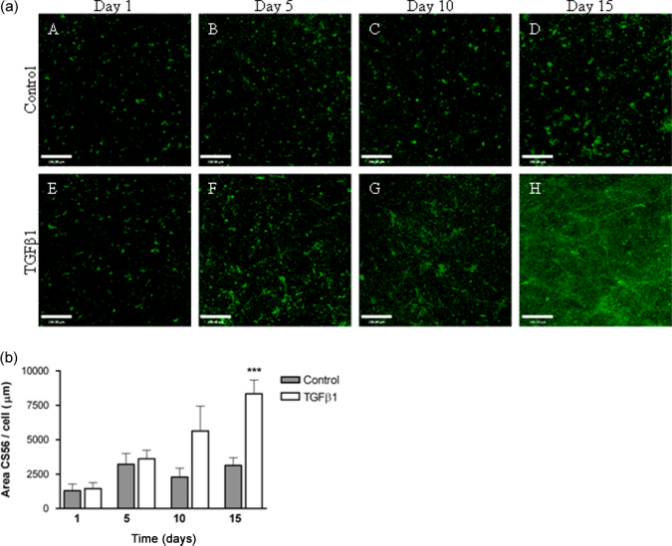
Area of astrocytic CSPG staining increases with TGFβ1-treatment. (a) Representative extended focus confocal images of control (A–D) and TGFβ1-treated (E–H) astrocytes over 15 days in culture stained for CS56, showing cell-associated staining and CSPG deposited by cells into the surrounding extracellular matrix. Scale bar = 200 μm. (b) Quantification of CSPG expression showed that area of staining significantly increased in TGFβ1-treated astrocytes after 15 days in culture compared to non-treated control cultures at the same time point (data are mean ± SEM normalized to cell number; *n* = 7; ***p < 0.001)

**Figure 6:**
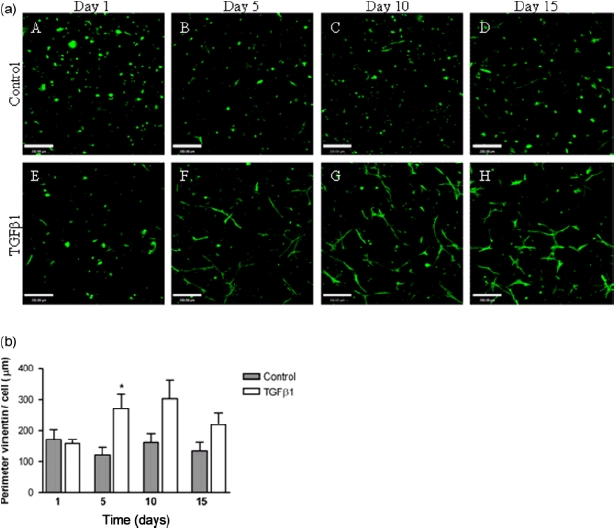
Perimeter of astrocytic vimentin staining increases with TGFβ1 treatment. (a) Representative extended focus confocal images of control (A–D) and TGFβ1-treated (E–H) astrocytes over 15 days in culture, stained for vimentin. Scale bar = 200 μm. (b) Quantification of vimentin staining showed that cell perimeter significantly increased in TGFβ1-treated astrocytes at 5 days in culture compared to non-treated control cultures at the same time point; although increases were seen at 10 and 15 days, these did not reach statistical significance (data are mean ± SEM normalized to cell number; *n* = 7; *p < 0.05)

**Figure 7:**
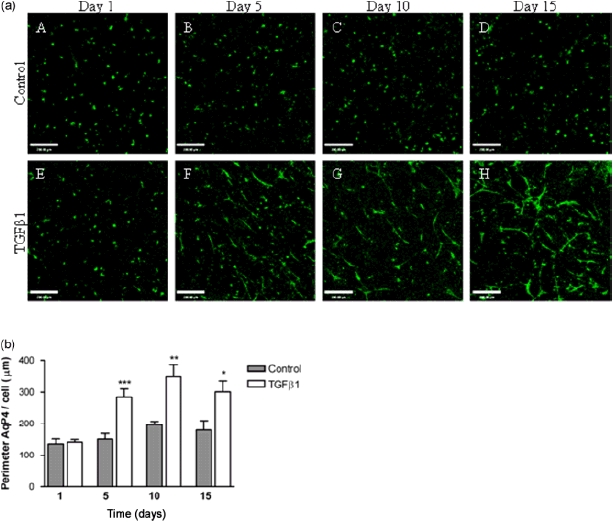
Perimeter of astrocytic aquaporin 4 staining increases with TGFβ1-treatment. (a) Representative extended focus confocal images of control (A–D) and TGFβ1-treated (E–H) astrocytes over 15 days in culture stained for AqP4. Scale bar = 200 μm. (b) Quantification of AqP4 staining showed that cell perimeter significantly increased in TGFβ1-treated astrocytes from 5 days in culture compared to non-treated control cultures at the same time point (data are mean ± SEM normalized to cell number; *n* = 7; *p > 0.05, **p > 0.01, ***p > 0.001)

TGFβ1 has been identified as one of the potential triggers of inhibitory glial scar formation following spinal cord injury (Silver and Miller, 2004) and it has previously been shown to induce specific alterations in astrocytes that are consistent with gliosis (Logan *et al.*, 1994). Following CNS injury *in vivo*, acute upregulation of TGFβ1 is detected within neurons, astrocytes and invading macrophages, and can additionally be found in astrocytes up to 1 year following injury (Buss *et al.*, 2008), suggesting a role in the formation and maintenance of the glial scar. Treatment of 3D astrocyte cultures with TGFβ1 for a 15 day period was utilized to recreate some aspects of a CNS injury environment. *In vivo*, the mature glial scar takes approximately 2 weeks to form, hence the 15 day time frame for this experiment. Analysis of immunofluorescence staining for GFAP, CSPGs, vimentin and AqP4 was performed to determine the extent of astrocyte activation. Staining for GFAP, vimentin and AqP4 revealed that control untreated cells over 15 days in culture showed very little change in morphology (Figures 4a, A-D; 6a, A-D; 7a, A-D). In addition, staining for CS56 revealed that there was very little change in expression and deposition of CSPGs in control untreated cultures over 15 days (Figure 5a A-D). However, with increasing time of TGFβ1-treatment, astrocytes appeared considerably more ramified compared to control cultures at the same time points when stained for GFAP, vimentin and AqP4 (Figures 4a, E-H; 6a, E-H; 7a, E-H) and there was more cell-associated CSPG staining and at day 15 more deposition of CSPGs in the gels (Figure 5a, E-H).

Quantification of changes in GFAP, vimentin and AqP4-positive cell morphology was assessed by measuring cell perimeter on extended-focus confocal micrographs. The area of staining was analysed for CS56 to determine cell-associated CSPG staining and that deposited by cells into the surrounding ECM. All data were normalized to the number of cells/field in order to correct for any potential cell proliferation over the 15 day time period of the experiment. The perimeter of GFAP, vimentin and AqP4 staining or the area of CS56 deposition did not significantly change in samples from non-treated control gels over the 15 days (Figures 4b, 5b, 6b, 7b). In astrocyte gels treated with TGFβ1, the perimeter of GFAP staining significantly increased at days 5, 10 and 15 (Figure 4b; *p* < 0.05), representative of the hypertrophy and ramification of these cells following treatment with TGFβ1. The area of CSPG staining increased significantly at day 15 in TGFβ1-treated astrocyte cultures (Figure 5b; *p* < 0.001), representative of the increased synthesis and release of CSPGs. The perimeter of vimentin staining significantly increased in TGFβ1-treated astrocytes after 5 days in culture, compared to non-treated control cultures at the same time point (Figure 6b; *p* < 0.05), although increases were seen at days 10 and 15, these did not reach statistical significance. In astrocyte gels treated with TGFβ1, the perimeter of AqP4 staining significantly increased at 5, 10 and 15 days (Figure 7b; *p* < 0.001, *p* < 0.01 and *p* < 0.05, respectively), again indicating a high degree of hypertrophy and ramification of these cells following cytokine treatment. The area of staining for GFAP, vimentin and AqP4 showed a similar pattern to perimeter measurements (data not shown).

Analysis of cell shape was performed on images from control and TGFβ1-treated astrocyte gels stained for GFAP. Cells that are circular have a value close to 1, whereas irregularly shaped cells have smaller values. Cell shape did not significantly differ between control cultures over the 15 day experiment (Figure 4c). However, GFAP-positive cells did become significantly more ramified (less circular) over time in TGFβ1-treated astrocyte cultures, compared to controls at the same time point (Figure 4c; *p* < 0.01 day 5, *p* < 0.001 day 10, and *p* < 0.0001 day 15).

### 3.5. Release of IL-6 by activated astrocytes

**Figure 8:**
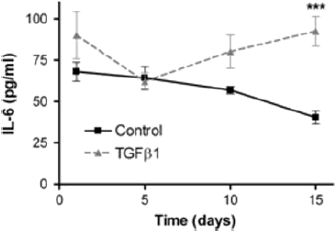
IL-6 release by astrocytes in 3D collagen gels. IL-6 was measured by ELISA of cell culture supernatants of control and TGFβ-1 treated astrocyte gel cultures at 1, 5, 10 and 15 days. IL-6 released from control cultures decreased over time, with a significant reduction detected after 15 days in culture (*p* < 0.01). IL-6 release from TGFβ1-treated astrocytes decreased at 5 days in culture, but then increased again over time and was significantly higher than control cultures at 15 days (data are mean ± SEM; *n* = 5; ***p < 0.001)

A key source of IL-6 in the injured spinal cord is astrocytes and its release is characteristic of their activation (Norris *et ah*, 1994; Van Wagoner and Benveniste, 1999; Farina *etal.*, 2007). IL-6 release from astrocytes was measured by ELISA of cell culture supernatants. Protein analysis revealed comparable levels of protein in all supernatant samples, thus normalization to protein content was not required and did not change the results. Levels of IL-6 release from control non-treated astrocytes decreased over time (Figure 8) and was significantly less at day 15 compared to day 1 in culture (*p* < 0.01). IL-6 release from TGFβ1-treated astrocytes decreased after 5 days in culture but increased again after 10 and 15 days. At day 15 levels were significantly higher than controls (*p* < 0.001; Figure 8).

## 4. Discussion

Here we report the development of a 3D *in vitro* model of astrogliosis that mimics many features of the glial scar environment within the CNS. Changing the cell culture dimension from conventional 2D monolayer to a 3D setting leads to downregulation of astrocyte activation markers, indicating that astrocytes in 3D are less reactive than their 2D counterparts, thus resembling the physiological *in vivo* situation and providing the first ‘ground state’ system in which triggers of astrogliosis can be investigated. This was validated using TGFβ1 treatment over 15 days, during which astrocytes adopted characteristics of the mature glial scar, with changes in cell morphology, expression of activation markers and secretion of cytokines associated with activation.

Substantial differences in gene and protein expression were observed in this study between 2D and 3D cultures, which is the first to undertake such a comparison using primary astrocytes. The 3D culture environment surrounds cells with ECM, providing nutritional and structural support from all directions, contrasting with a 2D environment where cells adhere on one side and receive nutrition from the other. Furthermore, cells adopt less constrained shapes in 3D gels, whereas in 2D culture they are forced to adjust to a flat surface (Lee *et al.*, 2008). Differences between other cell types in 2D and 3D environments have been noted, associated in particular with cell morphology and gene and protein expression (Pedersen and Swartz, 2005). A comprehensive study using neuroblastoma cells identified 1766 genes that were significantly differentially expressed between 3D and 2D culture (regardless of whether collagen or matrigel was the substrate), including those involved in cytoskeletal reorganization, ECM, metabolism and signalling (Li *et al.*, 2007), thus highlighting the impact of cell culture substrate geometry. In addition to spatial constraints, differences observed between 2D and 3D cultures are likely to reflect differences in mechanical cues, particularly stiffness of the matrix. Physical and mechanical cues are known to affect a multitude of cell functions, such as adhesion, proliferation, migration, differentiation and morphology (Li and Hoffman-Kim, 2008). In particular, stiffness sensing in astrocytes has been reported, with cells on soft gels showing small and round morphologies, similar to those seen in our 3D experiments, with few stress fibres. In contrast, on stiffer gel surfaces astrocytes showed significantly increased cell area, with highly spread morphologies and more stress actin fibres, typical of reactive cultured glia (Georges *et al.*, 2006) and similar to astrocytes seen in conventional 2D monolayer cultures. In our system, astrocytes at the edges of 3D cultures, where they were in contact with the stiff plastic, showed elevated GFAP levels and appeared more ramified, consistent with this previous suggestion that substrate stiffness increases astrocyte reactivity.

The glial scar *in vivo* is a chemical and physical barrier, involving soluble factors released by cells, molecules expressed on the surface of cells, changes in ECM composition and changes in the shape and size of the various cells involved (Silver and Miller, 2004; Yiu and He, 2006; Fitch and Silver, 2008). In terms of timing, cell morphology and expression of markers, the response of astrocytes to TGFβ1 treatment in our 3D system is similar to that seen *in vivo.* This suggests that TGFβ1 alone can be sufficient to induce astrogliosis, without the requirement of other cellular components or paracrine effects. This system also lends itself to analysis of cytokine secretion from astrocytes. Astrocytes are a key source of IL-6 in the injured spinal cord and its release is characteristic of their activation. It is unlikely in this model that IL-6 will positively feed back to initiate further astrocyte activation, since this would require the presence of the soluble IL-6 receptor, not produced by these cells (Van Wagoner and Benveniste, 1999; Van Wagoner *et al.*, 1999).

There are several cell culture models that have attempted to recreate the complex environment of the adult CNS, specifically glial scar, in three dimensions. One of the first was by Fawcett *et al.* (1989), who investigated the growth of axons in cultures created by packing astrocytes or Schwann cells into 0.5 mm i.d. cellulose ester tubes, then investigating the inhibition of neuronal growth in the 3D astrocyte environment. It is important to use 3D culture models to investigate the inhibition of neuronal growth, since in monolayers neurons are able to grow over the top of astrocyte cultures (Sorensen *et al.*, 2007). However, one potential drawback of the tube approach for modelling astrocyte behaviour is that, since cells are in contact with the curved 2D surface inside the tube, they are constrained from adopting a true 3D phenotype. Hydrogel systems are thus more appropriate for many situations because they create a 3D microenvironment in which cells are not subjected to mechanical cues, such as those that exist on the inner surfaces of tubes or within the pores of stiff scaffold materials (Brown and Phillips, 2007; East and Phillips, 2008). Furthermore, they enable mechanical cues to be isolated from chemical cues, in order to explore their effects on cells (Cullen *et al.*, 2007) and provide an intermediate degree of complexity between *in vivo* studies and 2D *in vitro* systems.

In summary, the present study first investigated differences between 2D and 3D primary astrocyte cultures and then, by exploiting the observation that astrocytes in 3D are less reactive than their monolayer counterparts, a system for investigating reactive astrogliosis was developed. This system recreated the key features of the response of astrocytes in the CNS following damage and has been shown to support a wide range of analytical techniques, including immunofluorescence image analysis, RT-PCR, time-lapse microscopy and ELISA. By further developing the system to incorporate multiple cell types, ECM molecules and mechanical response investigations, our future aim is to utilize this model as an *in vitro* test-bed in order to improve tissue-engineered CNS repair devices. Revealing the characteristics of astrocytes in this system provides new tools and opportunities for neuroscientists to explore the cell biology underlying *in vivo* observations, informing the development of a new generation of models that combine the accessibility of cell culture with the relevant spatial environment of living tissue.

## Supporting information on the internet

The following supporting information may be found in the online version of this article.

2D astrocyte time-lapse movie.

3D astrocyte time-lapse movie.
